# Radiologic Identification of Pathologic Tumor Invasion in Patients With Lung Adenocarcinoma

**DOI:** 10.1001/jamanetworkopen.2023.37889

**Published:** 2023-10-16

**Authors:** Ting Ye, Haoxuan Wu, Shengping Wang, Qiao Li, Yajia Gu, Junjie Ma, Jihong Lin, Mingqiang Kang, Bin Qian, Hong Hu, Yang Zhang, Yihua Sun, Yawei Zhang, Jiaqing Xiang, Yuan Li, Xuxia Shen, Zezhou Wang, Haiquan Chen

**Affiliations:** 1 Department of Thoracic Surgery and State Key Laboratory of Genetic Engineering, Fudan University Shanghai Cancer Center, Shanghai, China; 2 Institute of Thoracic Oncology, Fudan University, Shanghai, China; 3 Department of Oncology, Shanghai Medical College, Fudan University, Shanghai, China; 4 Department of Radiology, Fudan University Shanghai Cancer Center, Shanghai, China; 5 Department of Thoracic Surgery, The Second Hospital of Liaocheng Affiliated to Shandong First Medical University, Linqing, Shandong Province, China; 6 Department of Thoracic Surgery, Fujian Medical University Union Hospital, Fuzhou, Fujian Province, China; 7 Department of Thoracic Surgery, Jiangdu People’s Hospital of Yangzhou, Yangzhou, Jiangsu Province, China; 8 Department of Pathology, Fudan University Shanghai Cancer Center, Shanghai, China; 9 Department of Cancer Prevention, Fudan University Shanghai Cancer Center, Shanghai, China

## Abstract

**Importance:**

It is currently unclear whether high-resolution computed tomography can preoperatively identify pathologic tumor invasion for ground-glass opacity lung adenocarcinoma.

**Objectives:**

To evaluate the diagnostic value of high-resolution computed tomography for identifying pathologic tumor invasion for ground-glass opacity featured lung tumors.

**Design, Setting, and Participants:**

This prospective, multicenter diagnostic study enrolled patients with suspicious malignant ground-glass opacity nodules less than or equal to 30 mm from November 2019 to July 2021. Thoracic high-resolution computed tomography was performed, and pathologic tumor invasion (invasive adenocarcinoma vs adenocarcinoma in situ or minimally invasive adenocarcinoma) was estimated before surgery. Pathologic nonadenocarcinoma, benign diseases, or those without surgery were excluded from analyses; 673 patients were recruited, and 620 patients were included in the analysis. Statistical analysis was performed from October 2021 to January 2022.

**Exposure:**

Patients were grouped according to pathologic tumor invasion.

**Main Outcomes and Measures:**

Primary end point was diagnostic yield for pathologic tumor invasion. Secondary end point was diagnostic value of radiologic parameters.

**Results:**

Among 620 patients (442 [71.3%] female; mean [SD] age, 53.5 [12.0] years) with 622 nodules, 287 (46.1%) pure ground-glass opacity nodules and 335 (53.9%) part-solid nodules were analyzed. The median (range) size of nodules was 12.1 (3.8-30.0) mm; 47 adenocarcinomas in situ, 342 minimally invasive adenocarcinomas, and 233 invasive adenocarcinomas were confirmed. Overall, diagnostic accuracy was 83.0% (516 of 622; 95% CI, 79.8%-85.8%), diagnostic sensitivity was 82.4% (192 of 233; 95% CI, 76.9%-87.1%), and diagnostic specificity was 83.3% (324 of 389; 95% CI, 79.2%-86.9%). For tumors less than or equal to 10 mm, 3.6% (8 of 224) were diagnosed as invasive adenocarcinomas. The diagnostic accuracy was 96.0% (215 of 224; 95% CI, 92.5%-98.1%), diagnostic specificity was 97.2% (210 of 216; 95% CI, 94.1%-99.0%); for tumors greater than 20 mm, 6.9% (6 of 87) were diagnosed as adenocarcinomas in situ or minimally invasive adenocarcinomas. The diagnostic accuracy was 93.1% (81 of 87; 95% CI, 85.6%-97.4%) and diagnostic sensitivity was 97.5% (79 of 81; 95% CI, 91.4%-99.7%). For tumors between 10 to 20 mm, the diagnostic accuracy was 70.7% (220 of 311; 95% CI, 65.3%-75.7%), diagnostic sensitivity was 75.0% (108 of 144; 95% CI, 67.1%-81.8%), and diagnostic specificity was 67.1% (112 of 167; 95% CI, 59.4%-74.1%). Tumor size (odds ratio, 1.28; 95% CI, 1.18-1.39) and solid component size (odds ratio, 1.31; 95% CI, 1.22-1.42) could each independently serve as identifiers of pathologic invasive adenocarcinoma. When the cutoff value of solid component size was 6 mm, the diagnostic sensitivity was 84.6% (95% CI, 78.8%-89.4%) and specificity was 82.9% (95% CI, 75.6%-88.7%).

**Conclusions and relevance:**

In this diagnostic study, radiologic analysis showed good performance in identifying pathologic tumor invasion for ground-glass opacity–featured lung adenocarcinoma, especially for tumors less than or equal to 10 mm and greater than 20 mm; these results suggest that a solid component size of 6 mm could be clinically applied to distinguish pathologic tumor invasion.

## Key Points

**Question:**

Can high-resolution computed tomography preoperatively identify pathologic tumor invasion for ground-glass opacity nodules?

**Findings:**

In this diagnostic study that analyzed 620 patients from 4 Chinese institutions, the diagnostic accuracy for pathologic invasive adenocarcinoma was 83.0%; diagnostic sensitivity was 82.4%, and diagnostic specificity was 83.3%.

**Meaning:**

These results suggest that radiologic analysis showed good performance in identifying pathologic tumor invasion for ground-glass opacity–featured lung adenocarcinoma.

## Teaser

This diagnostic study investigates the diagnostic value of high-resolution computed tomography for identifying pathologic tumor invasion for ground-glass opacity featured lung tumors.

## Introduction

Pulmonary ground-glass opacity (GGO) nodules are detected increasingly with the application of high-resolution computed tomography (HRCT).^1,2^ Persistent GGO nodules often indicate preinvasive or invasive lung adenocarcinomas, which need close follow-up or surgical resection.^3^ In 2011, the International Association for the Study of Lung Cancer, American Thoracic Society, and European Respiratory Society recommended a new classification system for lung adenocarcinoma, in which lung adenocarcinoma was mainly classified as adenocarcinoma in situ (AIS), minimally invasive adenocarcinoma (MIA), and invasive adenocarcinoma (IAD). IADs were further classified based on the predominant histologic subtypes including lepidic, acinar, papillary, micropapillary, and solid patterns.^4^ The World Health Organization (WHO) endorsed this new classification of lung adenocarcinoma in 2015 and updated it in 2021.^5,6^ According to previous studies, patients with invasive adenocarcinoma vs AIS or MIA had quite different surgical outcomes.^7,8^ Two recent studies indicated that the 10-year postoperative disease-specific survival of patients with AIS or MIA was 100%,^9,10^ whereas patients with IAD had a worse disease-specific survival rate.^11^

Preoperative pathologic diagnosis for AIS or MIA cannot be made by biopsy, so radiologic evaluation, especially HRCT, has been clinically used to estimate the pathologic tumor invasion in order to identify surgical candidates for limited resection.^5,12,13^ According to the current WHO classification, whether HRCT could predict the pathologic tumor invasion for GGO tumors, especially distinguish AIS or MIA from IAD, was unclear. To answer this question, we performed this prospective, multicenter diagnostic study (Eastern Cooperative Thoracic Oncology Projects [ECTOP] 1008) to evaluate the diagnostic yield of HRCT in identifying pathologic tumor invasion (AIS or MIA vs IAD) for patients with GGO-featured lung cancer.

## Methods

This diagnostic study was conducted at 4 Chinese medical centers from November 2019 to July 2021. The protocol was approved by the institutional review boards at all 4 medical centers (Fudan University Shanghai Cancer Center, The Second Hospital of Liaocheng affiliated to Shandong First Medical University, Fujian Medical University Union Hospital, and Jiangdu People’s Hospital of Yangzhou). All enrolled patients provided written informed consent. This study (ECTOP-1008) was registered at ClinicalTrials.gov (NCT04165759) and was reported following the Standards for Reporting of Diagnostic Accuracy (STARD) reporting guideline.

The eligibility criteria were as follows: (1) suspicious malignant GGO nodules on HRCT scan; (2) clinical stage IA; (3) simultaneously no more than 3 nodules; (4) follow-up period at least 3 months; and (5) age ranging from 15 to 85 years. The exclusion criteria included: (1) pathological nonadenocarcinoma; (2) pathological benign diseases; and (3) patients who did not receive a surgical procedure. The primary end point of this study was the diagnostic yield of pathologic tumor invasion evaluated by HRCT. The secondary end point was diagnostic value of radiologic parameters on HRCT for pathologic tumor invasion of GGO-featured lung adenocarcinoma.

It was expected that the diagnostic sensitivity of pathologic invasive adenocarcinoma by HRCT for GGO nodules would be 80%. A minimum sample size of 607 participants would be required for the estimation of sensitivity with a 2-sided 95% CI width equal to 0.1 when the ratio of invasive adenocarcinoma for GGO nodules was 45% according to the previous study^11^ and considering a dropout rate of 10%.

All patients enrolled in this study received routine HRCT or target scans. All patients were trained to breathe before the scan. Patients were examined in the supine position and with deep inspiration breath-hold. The routine HRCT was obtained from the apex of the lung to the adrenal gland before treatment. The routine HRCT scan parameters were as follows: voltage, 120 kV; tube current, 250 mA; field of view, 400 mm; reconstruction slice thickness, 1 mm; interval, 1 mm; single collimation width, 0.5 mm; pixel spacing, 0.74 mm; and image matrix, 512 × 512.

Radiologic evaluation was performed by each participating center and consisted of 3 experienced thoracic surgeons or chest radiologists. GGO nodule was defined as a radiologic lesion showing a hazy opacity without blocking underlying pulmonary vessels or bronchial structures on HRCT scan.^3^ Pure GGO nodule was defined as a nodule without a solid component, and part-solid nodule was defined as a nodule with both GGO and solid component (eFigure 1 in Supplement 1).^14^ The maximum diameter on the single largest axial dimension measured on the lung window was recorded for the size of solid component and the whole nodule. When the solid component was irregular or multiple, multiple plane reconstruction was used, and only the largest was analyzed. Radiologic parameters that were measured and recorded included: (1) whole tumor size; (2) solid component size; (3) shape: classified into round or oval and polygonal or irregular; (4) margin: classified into smooth and lobulated or spiculated; (5) tumor-lung interface: classified into clear and unclear; (6) presence of bubble lucency; (7) presence of air bronchogram; and (8) presence of pleural indentation (eFigure 2 in Supplement 1). Radiologic noninvasive adenocarcinoma (estimated to be pathologic AIS or MIA) or radiologic invasive adenocarcinoma (estimated to be pathologic IAD) were evaluated before surgery. Radiologic criteria for noninvasive adenocarcinoma were: (1) pure GGO nodules with maximal diameter less than or equal to 2 cm and (2) GGO predominant nodules with solid component size less than 6 mm. Radiologic criteria for invasive adenocarcinoma were: (1) pure GGO nodules greater than 2 cm; (2) solid predominant nodules; and (3) GGO predominant nodules with solid component size greater than or equal to 6 mm. When different opinions occurred, agreement was reached through discussion among the 3 evaluators.

Sublobar resection (wedge resection or segmentectomy) or lobectomy was mainly selected according to the radiologic features including tumor location, tumor size, solid component size, and intraoperative frozen section. Postoperative pathologic diagnosis was made according to the 2015 WHO Classification of Tumors of the Lung, Pleura, Thymus, and Heart.^5^ Lung adenocarcinoma was classified as AIS, MIA, and IAD.^15^ IADs were further divided into lepidic predominant adenocarcinoma, acinar predominant adenocarcinoma, papillary predominant adenocarcinoma, micropapillary predominant adenocarcinoma, solid predominant adenocarcinoma, and invasive mucinous adenocarcinoma. The predominant subtype was defined as the pattern with the largest percentage (not necessarily 50% or higher). Pathologic staging was according to the eighth edition of the tumor, node, and metastasis (TNM) classification of lung cancer.^16^

### Statistical Analysis

The data were reported as number (%) for categorical variables. Continuous variables were described as mean (SD) or median (range). Diagnostic accuracy was defined as the proportion of patients whose radiologic estimation was consistent with pathologic diagnosis. Diagnostic sensitivity was defined as the proportion of patients with radiologic invasive adenocarcinoma in patients with pathologic IADs. Diagnostic specificity was defined as the proportion of patients with radiologic noninvasive adenocarcinoma in patients with pathologic AIS or MIA. A logistic regression model was used to identify radiologic variables for pathologic IAD. Factors with *P* < .05 in univariable analysis were then analyzed by multivariable analysis. Two-sided *P* < .05 was considered statistically significant. All statistical analyses were performed using R version 4.0.2 and R Studio version 1.3.1073 (R Project for Statistical Computing) from October 2021 to January 2022.

## Results

**Figure 1.**
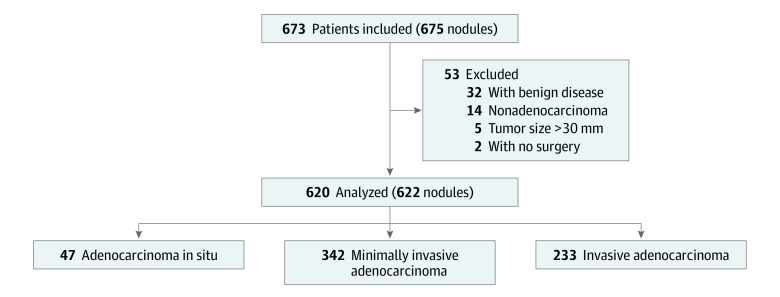
Flowchart of Patient Selection

Between November 2019 and July 2021, a total of 673 patients (475 [70.4%] female; mean [SD] age, 53.3 [12.0] years) with 675 nodules were recruited in this study. Two patients did not receive a surgical procedure, 5 patients had a tumor with maximal diameter greater than 30 mm, 32 patients were pathologically diagnosed as having benign diseases, 12 patients were diagnosed as having atypical adenomatous hyperplasia, and 2 patients were diagnosed as having mucosa-associated lymphoid tissue. After these exclusions, 620 patients with 622 nodules were analyzed (Figure 1). There were 442 female (71.3%) and 178 male (28.7%) patients. The mean (SD) age was 53.5 (12.0) years (range, 16-83 years). There were 287 pure GGO nodules (46.1%) and 335 part-solid nodules (53.9%). Median (range) radiologic tumor size was 12.1 (3.8-30.0) mm, and median (range) solid component size was 2.5 (0-26.0) mm. There were 322 (51.7%) wedge resections, 179 (28.8%) segmentectomies, and 121 (19.5%) lobectomies performed. Pathologically, 47 (7.6%) AISs, 342 (55.0%) MIAs, and 233 (37.4%) IADs were confirmed. No N1 or N2 diseases were detected (Table 1).

**Table 1.** Clinicopathologic Characteristics of 620 Patients With 622 Nodules

Characteristics	Patients or nodules, No. (%)
Sex	
Female	442 (71.3)
Male	178 (28.7)
Age, mean (SD), y	53.5 (12.0)
Smoking history, ever	102 (16.5)
Operative procedure	
Lobectomy	121 (19.5)
Segmentectomy	179 (28.8)
Wedge resection	322 (51.7)
Tumor location	
LUL	158 (25.4)
LLL	96 (15.4)
RUL	216 (34.7)
RML	43 (7.0)
RLL	109 (17.5)
Radiologic parameters	
Radiologic tumor size, mm	
≤10	224 (36.0)
10 to ≤20	311 (50.0)
>20	87 (14.0)
Solid component size, median (range), mm	2.5 (0-26.0)
Shape	
Round/oval	244 (39.2)
Polygonal/irregular	378 (60.8)
Margin	
Smooth	244 (39.2)
Lobulated/spiculated	378 (60.8)
Tumor-lung interface	
Clear	476 (76.5)
Unclear	146 (23.5)
Bubble lucency	
Absent	267 (42.9)
Present	355 (57.1)
Air bronchogram	
Absent	313 (50.3)
Present	309 (49.7)
Pleural indentation	
Absent	409 (65.8)
Present	213 (34.2)
Pathologic characteristics	
Pathological diagnosis	
AIS	47 (7.6)
MIA	342 (55.0)
IAD	233 (37.4)
Histological predominant subtypes	
LPA	48 (20.6)
APA	130 (55.8)
PPA	52 (22.3)
MPA	0 (0)
SPA	1 (0.4)
IMA	2 (0.9)
Pathologic tumor size, median (range), mm	10.0 (3.0-35.0)
Pathological stage	
0	47 (7.5)
IA1	382 (61.4)
IA2	141 (22.7)
IA3	19 (3.1)
IB	33 (5.3)

Abbreviations: AIS, adenocarcinoma in situ; APA, acinar predominant adenocarcinoma; IAD, invasive adenocarcinoma; IMA, invasive mucinous adenocarcinoma; LLL, left lower lobe; LPA, lepidic predominant adenocarcinoma; LUL, left upper lobe; MIA, minimally invasive adenocarcinoma; MPA, micropapillary predominant adenocarcinoma; PPA, papillary predominant adenocarcinoma; RLL, right lower lobe; RML, right middle lobe; RUL, right upper lobe; SPA, solid predominant adenocarcinoma.

Overall, the radiologic diagnostic accuracy for pathologic tumor invasion was 83.0% (516 of 622; 95% CI, 79.8%-85.8%). The radiologic diagnostic sensitivity for pathologic tumor invasion was 82.4% (192 of 233; 95% CI, 76.9%-87.1%), and the specificity was 83.3% (324 of 389; 95% CI, 79.2%-86.9%). According to the different tumor sizes, for tumors with maximal diameter less than or equal to 10 mm, the diagnostic accuracy was 96.0% (215 of 224; 95% CI, 92.5%-98.1%), the diagnostic sensitivity was 62.5% (5 of 8; 95% CI, 24.5%-91.5%), and the diagnostic specificity was 97.2% (210 of 216; 95% CI, 94.1%-99.0%). For tumors with the maximal diameter between 10 to 20 mm, the diagnostic accuracy was 70.7% (220 of 311; 95% CI, 65.3%-75.7%), the diagnostic sensitivity was 75.0% (108 of 144; 95% CI, 67.1%-81.8%), and the diagnostic specificity was 67.1% (112 of 167; 95% CI, 59.4%-74.1%). For tumors with maximal diameter greater than 20 mm, the diagnostic accuracy was 93.1% (81 of 87; 95% CI, 85.6%-97.4%), the diagnostic sensitivity was 97.5% (79 of 81; 95% CI, 91.4%-99.7%), and the diagnostic specificity was 33.3% (2 of 6; 95% CI, 4.3%-77.7%) (Table 2).

**Table 2.** Radiologic Factors and Pathologic Diagnosis

Radiologic identification	Pathologic diagnosis	
	Noninvasive	Invasive, % (95% CI)
**Overall**		
Noninvasive, No.	324	41
Invasive, No.	65	192
Accuracy	NA	83.0 (79.8-85.8)
Sensitivity	NA	82.4 (76.9-87.1)
Specificity	NA	83.3 (79.2-86.9)
**Tumor size ≤10 mm**		
Noninvasive, No.	210	3
Invasive, No.	6	5
Accuracy	NA	96.0 (92.5-98.1)
Sensitivity	NA	62.5 (24.5-91.5)
Specificity	NA	97.2 (94.1-99.0)
**Tumor size 10 mm to ≤20 mm**		
Noninvasive, No.	112	36
Invasive, No.	55	108
Accuracy	NA	70.7 (65.3-75.7)
Sensitivity	NA	75.0 (67.1-81.8)
Specificity	NA	67.1 (59.4-74.1)
**Tumor size >20 mm**		
Noninvasive, No.	2	2
Invasive, No.	4	79
Accuracy	NA	93.1 (85.6-97.4)
Sensitivity	NA	97.5 (91.4-99.7)
Specificity	NA	33.3 (94.3-77.7)

Abbreviation: NA, not applicable.

Radiologic factors on HRCT were evaluated for pathologic invasive adenocarcinoma. The independent radiologic factors were larger tumor size (OR, 1.28; 95% CI, 1.18-1.39; *P* < .001), larger solid component size (OR, 1.31; 95% CI, 1.22-1.42; *P* < .001), and lobulated or spiculated margin (OR, 5.14, 95% CI, 1.69-17.08; *P* = .005) on multivariable analysis (Table 3).

**Table 3.** Univariable and Multivariable Analysis for Radiologic Parameters in Identifying Pathologic Tumor Invasiveness

Variable	Univariable OR (95% CI)	*P* value	Multivariable OR (95% CI)	*P* value
Tumor size	1.51 (1.42-1.61)	<.001	1.28 (1.18-1.39)	<.001
Solid component size	1.54 (1.45-1.65)	<.001	1.31 (1.22-1.42)	<.001
Shape				
Round/oval	1 [Reference]	<.001	1 [Reference]	.59
Polygonal/irregular	24.74 (14.16-46.97)		0.74 (0.25-2.17)	
Margin				
Smooth	1 [Reference]	<.001	1 [Reference]	.005
Lobulated/spiculated	33.67 (18.19-69.74)		5.14 (1.69-17.08)	
Tumor-lung interface				
Clear	1 [Reference]	<.001	1 [Reference]	.27
Unclear	3.09 (2.11-4.54)		0.71 (0.38-1.31)	
Bubble lucency				
Absent	1 [Reference]	<.001	1 [Reference]	.48
Present	5.77 (3.96-8.56)		1.24 (0.67-2.28)	
Air bronchogram				
Absent	1 [Reference]	<.001	1 [Reference]	.32
Present	8.56 (5.87-12.68)		1.37 (0.74-2.53)	
Pleural indentation				
Absent	1 [Reference]	<.001	1 [Reference]	.08
Present	5.49 (3.85-7.90)		1.65 (0.94-2.89)	

Abbreviation: OR, odds ratio.

**Figure 2.**
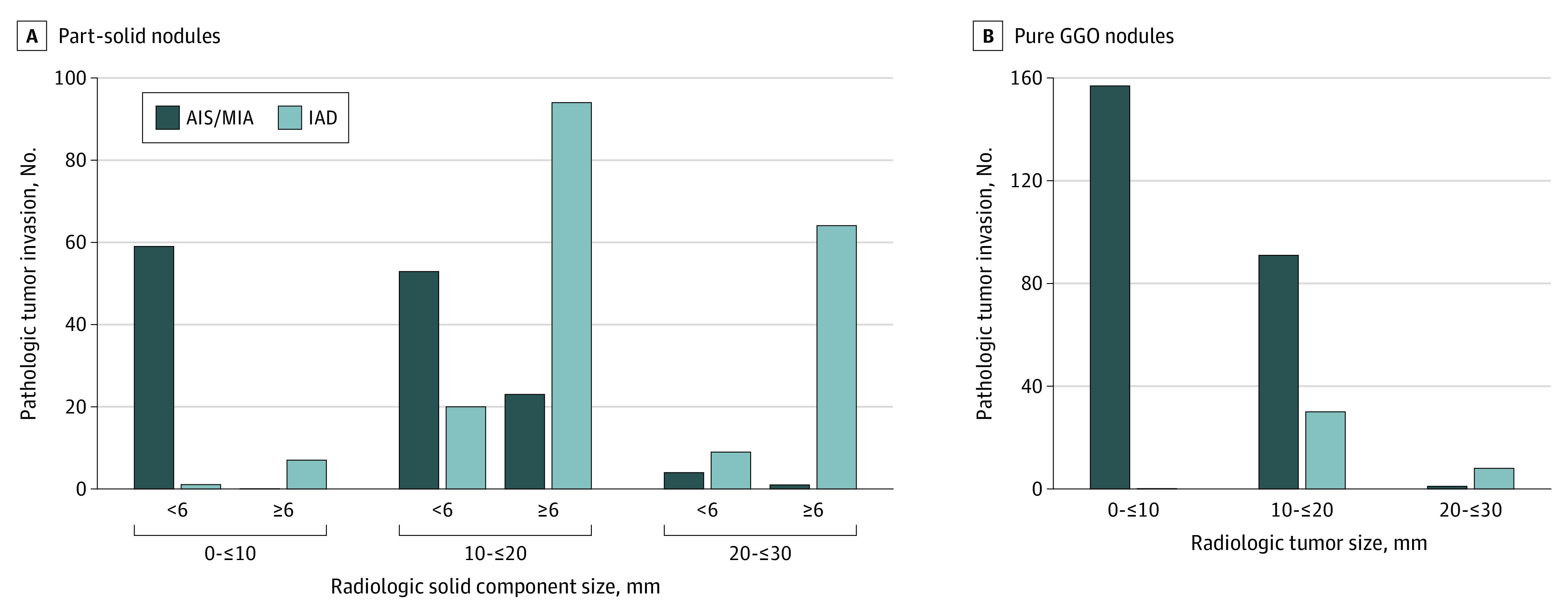
Pathologic Tumor Invasion A, The pathologic tumor invasion for part-solid nodules in different radiologic tumor size when adopting the radiologic solid component size 6 mm as cutoff value. B, The pathologic tumor invasion for pure ground-glass opacity (GGO) nodules in different radiologic tumor size. AIS indicates adenocarcinoma in situ; IAD, invasive adenocarcinoma; MIA, minimally invasive adenocarcinoma.

Accordingly, tumor size was an important factor for distinguishing pathologic AIS or MIA from IAD. For 224 tumors with maximal diameter less than or equal to 10 mm, there were only 8 IADs (3.6%); for 87 tumors with maximal diameter greater than 20 mm, there were only 6 AISs or MIAs (6.9%); for 311 tumors with a maximal diameter between 10 to 20 mm, there were 144 IADs (46.3%) and 167 MIAs (53.7%). In addition, the solid component size was the other important factor for distinguishing pathologic AIS or MIA from IAD. Especially for part-solid nodules, a solid component size of 6 mm was recognized as the optimal cutoff value. Overall, 82.9% (116 of 140; 95% CI, 75.6%-88.7%) of AISs or MIAs had a solid component less than 6 mm, whereas 84.6% (165 of 195; 95% CI, 78.8%-89.4%) of IADs had a solid component size of at least 6 mm (Figure 2A and eFigure 3 in Supplement 1). In addition, the solid component size of 6 mm performed well in distinguishing AIS or MIA from IAD in different tumor size groups (tumor size ≤10 mm: sensitivity, 87.5% [7 of 8; 95% CI, 47.3%-99.7%]; specificity, 100% [59 of 59; 95% CI, 93.9%-100.0%]; tumor size 10 mm to ≤20 mm: sensitivity, 82.5% [94 of 114; 95% CI, 74.2%-88.9%]; specificity, 69.7% [53 of 76; 95% CI, 58.1%-79.8%]; tumor size >20 mm: sensitivity, 87.7% [64 of 73; 95% CI, 77.9%-94.2%]; specificity, 80.0% [4 of 5; 95% CI, 28.4%-99.5%]) (eTable in Supplement 1).

For pure GGO nodules, the efficacy of HRCT for distinguishing pathologic AIS or MIA from IAD varied greatly according to different tumor sizes. For tumors with maximal diameter less than or equal to 10 mm, there were 157 pure GGO nodules, and all of them were AISs or MIAs (157 of 157 [100%]); for tumors with the maximal diameter between 10 to 20 mm, there were 121 pure GGO nodules, and 30 were IADs (24.8%), whereas 91 were AISs or MIAs (75.2%); for tumors with maximal diameter greater than 20 mm, there were 9 pure GGO nodules, and only 1 was AIS or MIA (11.1%), and the other 8 were IADs (88.9%) (Figure 2B).

Notably, for 233 pathologic-confirmed IADs, there were 48 lepidic predominant adenocarcinomas (20.6%), 130 acinar predominant adenocarcinomas (55.8%), 52 papillary predominant adenocarcinomas (22.3%), 1 solid predominant adenocarcinoma (0.4%), and 2 mucinous adenocarcinomas (0.9%). Only 4 tumors (1.7%) with vascular or lymphatic invasion were identified. Especially for 38 IADs with radiologic pure GGO, there were 12 lepidic predominant adenocarcinomas, 16 acinar predominant adenocarcinomas, and 10 papillary predominant adenocarcinomas. No vascular or lymphatic invasion was detected (eFigure 4 in Supplement 1).

## Discussion

Several studies have focused on HRCT to identify pathologic tumor invasion, but few of them were prospective.^8,11,12^ In 2002, Suzuki and colleagues^12^ performed the only prospective study at the time to define the radiologic noninvasive lung cancer (JCOG 0201). In 2011, they found that tumor size less than or equal to 2 cm and consolidation-to-tumor (C/T) ratio less than or equal to 0.25 on HRCT could identify pathologic noninvasive adenocarcinoma in clinical IA lung cancer. The diagnostic specificity was 98.7% and the sensitivity was 16.2%.^12^ In 2013, they analyzed the postoperative survival data of patients with radiologic noninvasive lung cancer, and both the 5-year disease-free survival and overall survival were 97.1%, which were favorable. However, patients with radiologic noninvasive tumors had similar survival rates compared with those with radiologic invasive tumors.^13^ Moreover, 2 recent studies indicated that for patients with AIS or MIA, the 10-year disease-specific survival was 100%,^9,10^ which was even higher. In addition, the definition of pathological noninvasive lung cancer in JCOG 0201, which was pN0 adenocarcinoma without vascular and lymphatic involvement on resected specimen, was quite different from the 2015 WHO classification of lung adenocarcinoma.^5^ According to JCOG 0804, which was a single-group study to confirm the efficacy of sublobar resection for radiologic noninvasive lung cancer defined by JCOG 0201, there were nearly 20% pathologic invasive adenocarcinomas for GGO nodules less than or equal to 2cm with C/T ratio less than or equal to 0.25.^17^ This definition failed to distinguish AIS or MIA from IAD, while patients with AIS or MIA compared with IAD had quite different postoperative survivals.^9,10,11^ Precise diagnosis between AIS or MIA and IAD is clinically important because overdiagnosis for AIS or MIA would result in overtreatment, and underdiagnosis for IAD would result in undertreatment. Sublobar resection is appropriate for AIS or MIA, whereas lobectomy or segmentectomy is the standard procedure for IAD.

Results of this study indicated that the tumor size and solid component size were the 2 most important factors for HRCT to identify pathologic tumor invasion. For tumors with a maximal diameter less than or equal to 10 mm, the diagnostic accuracy was 96.0%, and the diagnostic specificity was 97.2%. There were only 8 IADs, and 7 had the solid component size greater than or equal to 6 mm. For tumors with the maximal diameter greater than 20 mm, the diagnostic accuracy was 93.1%, and the diagnostic sensitivity was 97.5%. There were only 6 MIAs, and 4 had the solid component size less than 6 mm; 1 was a pure GGO nodule, and the other 1 had a solid component size greater than 6 mm. For tumors with maximal diameter between 10 to 20 mm, when the solid component size of 6 mm was used, the sensitivity was 82.5% and the specificity was 69.7%. For the tumors between 10 to 20 mm, further investigation, for example artificial intelligence (AI), especially machine learning algorithms based on radiomic features, might be needed to improve the diagnostic yield. It was unknown whether the misdiagnosis for these tumors could affect the patients’ prognosis because the survival rates of these patients needed a long follow-up period. Currently, intraoperative pathologic evaluation might be still necessary when limited resection was considered.^18^

Radiologic parameters were widely evaluated to identify pathologic tumor invasiveness for GGO-featured lung adenocarcinomas.^8,11,19,20^ Most studies focused on tumor size and C/T ratio. In 2015, Kudo and colleagues^19^ indicated that higher C/T ratio and larger tumor consolidation size were associated with pathologic IAD in part-solid lung cancers. In addition, a previous study also found that larger solid component size and tumor size were associated with pathologic IAD for GGO tumors.^11^ Compared with C/T ratio, the solid component size might be a more direct and practical variable. In this study, for tumors less than 1 cm, all IADs had a larger solid component size, and this made the C/T ratio very high. Contrarily, for tumors larger than 2 cm, tumors with a solid component size of 1 to 2 mm could be pathologic IAD, and this made the C/T ratio very low. Moreover, considering the solid component size is a continuous variable, identification of an appropriate cutoff value is necessary for clinical application. In this study, we found that the solid component size of 6 mm was an ideal cutoff value because it could provide both the favorable diagnostic sensitivity and specificity for part-solid tumors.

Interestingly, the extent of tumor invasion of radiologic pure GGO tumors varied greatly according to the different tumor sizes in this study. For tumors greater than or equal to 10 mm, all 157 pure GGO tumors were AIS or MIA (157/157; 100%); for tumors between 10 to 20 mm, 91 pure GGO tumors were AIS or MIA (91 of 121; 75.2%). However, for 9 pure GGO tumors greater than 20 mm, only 1 was AIS or MIA (1 of 9; 11.1%), while the other 8 were IADs (8 of 9; 88.9%). It could be speculated that for pure GGO tumors less than or equal to 20 mm, GGO tended to be pathologically lepidic growth, whereas for pure GGO tumors greater than 20 mm, GGO tended to be pathologically nonlepidic but invasive patterns. This finding challenges the traditional concept that GGO always corresponds to a pathologic lepidic pattern.^21^ Therefore, further radiologic-pathologic correlation analysis of GGO and solid component is necessary for better understanding of the pathology and the radiologic features for subsolid lung tumors.

### Limitations

There were limitations in this study. First, measurement of the radiologic parameters such as tumor margin, tumor-lung interface, and pleural indentation was sometimes subjective. Second, no long-term survival data of these patients was currently available because the end points of the study were not designed to address this issue.

## Conclusions

This study found good diagnostic accuracy, sensitivity, and specificity (83.0%, 82.4% and 83.3%) of HRCT to preoperatively identify pathologic tumor invasion for GGO-featured lung adenocarcinoma. The radiologic tumor size and solid component size were the 2 most important factors. For tumors less than or equal to 10 mm and greater than 20 mm, the diagnostic yields were better. A solid component size of 6 mm on HRCT could be an optimal cutoff value to distinguish pathologic AIS or MIA from IAD for clinical application.

## Notes

Supplement 1. **eFigure 1.** Representative Images of Ground Glass Opacity Nodule Shown on Routine High Resolution Computed Tomography **eFigure 2.** Representative Images of Radiologic Features **eFigure 3.** The Sensitivity and Specificity for the Solid Component Sizes in Identifying Pathologic Invasive Adenocarcinoma for Part-Solid Nodules **eFigure 4.** The Pathologic Characteristics of the Pathologic Invasive Adenocarcinomas in This Study **eTable.** Cut-Off Values of Solid Component Size for Identifying Invasive Adenocarcinoma in Part-Solid Nodules

Supplement 2.
Data Sharing Statement
