# Supplementary material for: Radiologic Identification of Pathologic Tumor Invasion in Patients With Lung Adenocarcinoma
Source: JAMA Netw Open. 2023 Oct 16;6(10):e2337889. doi: 10.1001/jamanetworkopen.2023.37889 (PMC10580106; doi:10.1001/jamanetworkopen.2023.37889)
Supplement: Supplement 1. — eFigure 1. Representative Images of Ground Glass Opacity Nodule Shown on Routine High Resolution Computed Tomography eFigure 2. Representative Images of Radiologic Features eFigure 3. The Sensitivity and Specificity for the Solid Component Sizes in Identifying Pathologic Invasive Adenocarcinoma for Part-Solid Nodules eFigure 4. The Pathologic Characteristics of the Pathologic Invasive Adenocarcinomas in This Study eTable. Cut-Off Values of Solid Component Size for Identifying Invasive Adenocarcinoma in Part-Solid Nodules [file jamanetwopen-e2337889-s001.pdf]

Supplemental Online Content

Ye T, Wu H, Wang S, et al. Radiologic identification of pathologic tumor invasion in patients with lung adenocarcinoma. *JAMA Netw Open.* 2023;6(10):e2337889. doi:10.1001/jamanetworkopen.2023.37889

eFigure 1. Representative Images of Ground Glass Opacity Nodule Shown on Routine High Resolution Computed Tomography

eFigure 2. Representative Images of Radiologic Features

eFigure 3. The Sensitivity and Specificity for the Solid Component Sizes in Identifying Pathologic Invasive Adenocarcinoma for Part-Solid Nodules

eFigure 4. The Pathologic Characteristics of the Pathologic Invasive Adenocarcinomas in This Study

eTable. Cut-Off Values of Solid Component Size for Identifying Invasive Adenocarcinoma in Part-Solid Nodules

This supplemental material has been provided by the authors to give readers additional information about their work.

© 2023 Ye T et al. *JAMA Network Open.*

eFigure 1. Representative Images of Ground Glass Opacity Nodule Shown on Routine High Resolution Computed Tomography

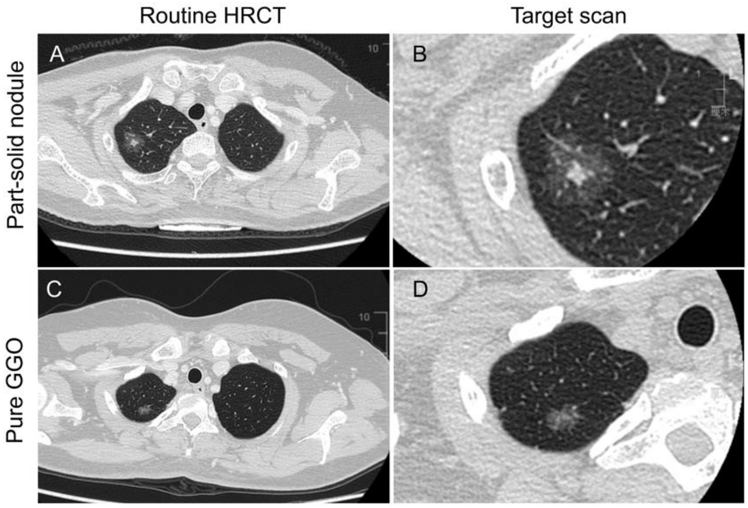

eFigure 1. Representative images of part-solid nodule and pure ground glass opacity (GGO) nodule shown on routine high resolution computed tomography (HRCT) scan and target scan.

© 2023 Ye T et al. *JAMA Network Open.*

eFigure 2. Representative Images of Radiologic Features

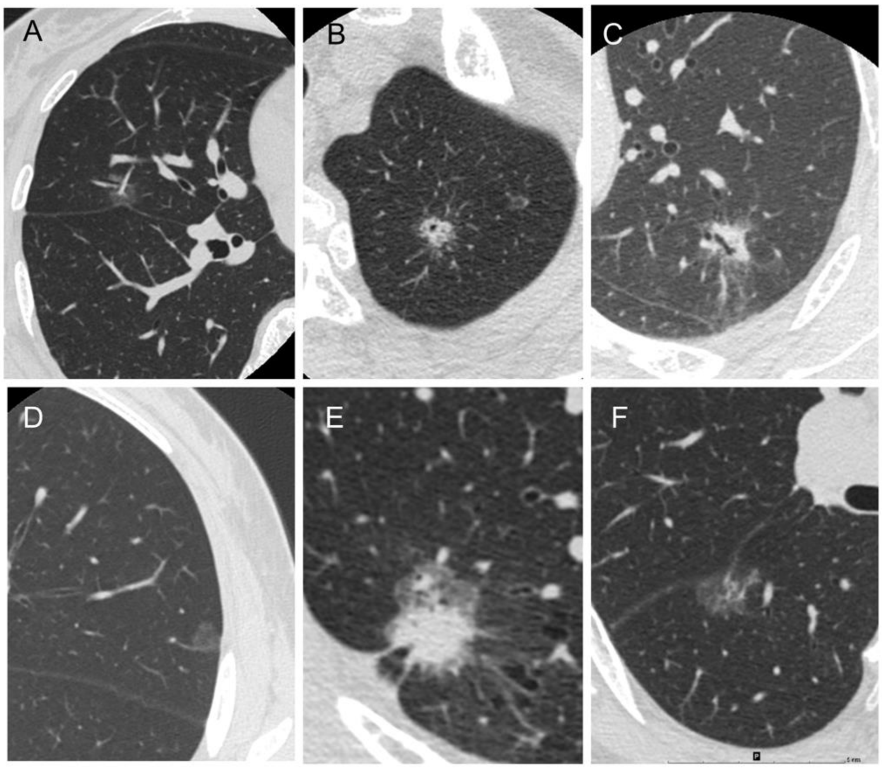

eFigure 2. Representative images of radiologic features. (A) A pure GGO nodule featured as presence of air bronchogram and pleural indentation. (B) A part-solid nodule featured as unclear tumor-lung interface, presence of bubble lucency and air bronchogram. (C) A part-solid nodule featured as unclear tumor-lung interface and presence of air bronchogram. (D) A pure GGO nodule featured as clear tumor-lung interface. (E) A part-solid nodule featured as lobulated and spiculated margin, presence of pleural indentation and air bronchogram. (F) A pure GGO nodule featured as lobulated margin, clear tumor-lung interface and presence of pleural indentation.

© 2023 Ye T et al. *JAMA Network Open.*

eFigure 3. The Sensitivity and Specificity for the Solid Component Sizes in Identifying Pathologic Invasive Adenocarcinoma for Part-Solid Nodules

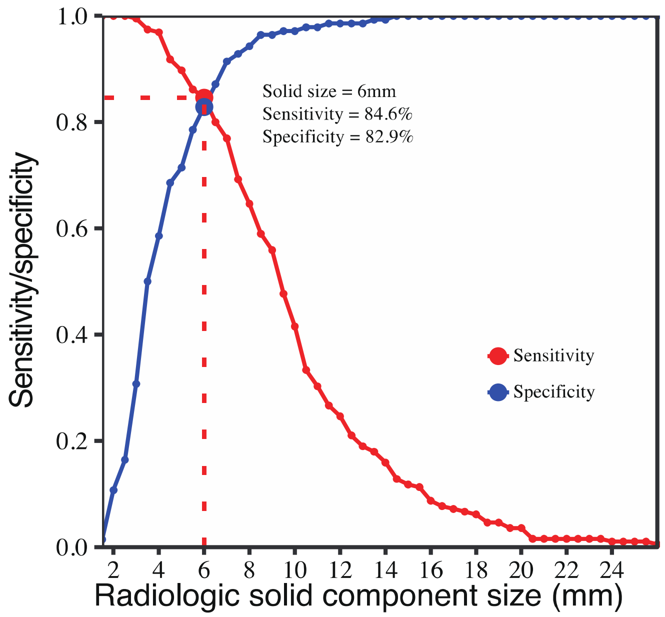

eFigure 3. The sensitivity and specificity for the solid component sizes in identifying pathologic invasive adenocarcinoma for part-solid nodules. A solid component size of 6mm was identified as the optimal cut-off value, with the sensitivity of 84.6% and the specificity of 82.9%.

© 2023 Ye T et al. *JAMA Network Open.*

eFigure 4. The Pathologic Characteristics of the Pathologic Invasive Adenocarcinomas in This Study

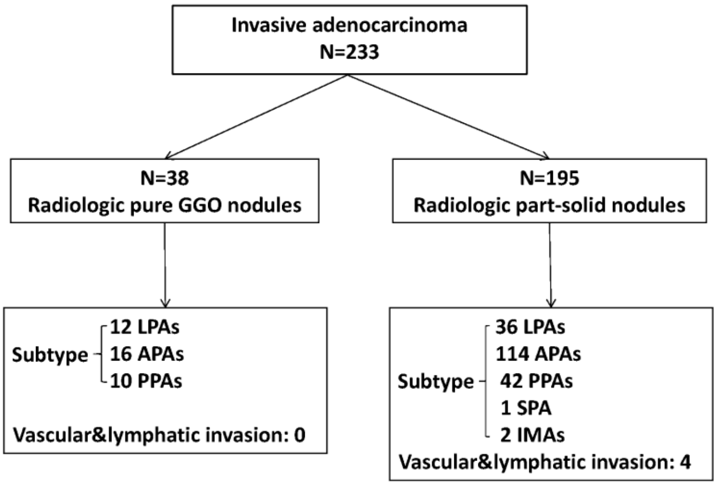

eFigure 4. The pathologic characteristics of the pathologic invasive adenocarcinomas in this study.

© 2023 Ye T et al. *JAMA Network Open.*

eTable . Cut-off values of solid component size for identifying invasive adenocarcinoma in part-solid nodules.

Solid component size (mm)	All tumors		Tumor size ≤10mm		10mm < Tumor size ≤ 20mm		Tumor size > 20mm	
	Sensitivity	Specificity	Sensitivity	Specificity	Sensitivity	Specificity	Sensitivity	Specificity
5	89.7%	71.4%	100.0%	93.2%	87.7%	55.3%	91.8%	60.0%
5.5	86.2%	78.6%	87.5%	100.0%	84.2%	63.2%	89.0%	60.0%
**6**	**84.6%**	**82.9%**	**87.5%**	**100.0%**	**82.5%**	**69.7%**	**87.7%**	**80.0%**
6.5	80.0%	87.1%	62.5%	100.0%	76.3%	77.6%	87.7%	80.0%
7	76.9%	91.4%	50.0%	100.0%	72.8%	85.5%	86.3%	80.0%

© 2023 Ye T et al. *JAMA Network Open.*
