# Supplementary material for: Radiologic Identification of Pathologic Tumor Invasion in Patients With Lung Adenocarcinoma
Source: JAMA Netw Open. 2023 Oct 16;6(10):e2337889. doi: 10.1001/jamanetworkopen.2023.37889 (PMC10580106; doi:10.1001/jamanetworkopen.2023.37889)
Supplement: Supplement 2. — Data Sharing Statement [file jamanetwopen-e2337889-s002.pdf]

Data Sharing Statement

Ye. Radiologic Identification of Pathologic Tumor Invasion in Patients With Lung Adenocarcinoma. *JAMA Netw Open*. Published October 16, 2023.
doi:10.1001/jamanetworkopen.2023.37889

Data
Data available: Yes
Data types: Participant data with identifiers
How to access data: Trial data relating to this publication could be disclosed to investigators who provide a written request, starting immediately and ending 3 years after publication. Data sharing requests should be directed to Dr. Haiquan Chen. Requests for data access and sharing for the trial should be emailed at hqchen1@yahoo.com.
When available: With publication

Supporting Documents
Document types: None

Additional Information
Who can access the data: Researchers whose proposed use of the data has been approved.
Types of analyses: Only for research purpose.
Mechanisms of data availability: Data sharing requests should be directed to Dr. Haiquan Chen. Requests for data access and sharing for the trial should be emailed at hqchen1@yahoo.com.
